# Spent Culture Medium miR-372-3p Is Associated with Blastocyst Morphology and Morphokinetic Dynamics in Human Embryos

**DOI:** 10.3390/diagnostics16172835

**Published:** 2026-09-03

**Authors:** Yu-Yang Hsiao, Hsuan-Wei Huang, Ni-Chin Tsai, Yu-Ju Lin, Hao-Ting Lien, Kuo-Chung Lan

**Affiliations:** 1 Department of Obstetrics and Gynecology, Kaohsiung Chang Gung Memorial Hospital, Chang Gung University College of Medicine, Kaohsiung 833, Taiwan; 2 Graduate Institute of Clinical Medicine Science, Chang Gung University College of Medicine, Taoyuan 333, Taiwan; 3 Department of Obstetrics and Gynecology, Jen-Ai Hospital, Taichung 483, Taiwan; 4 Kaohsiung Municipal Ta-Tung Hospital, Kaohsiung 801, Taiwan; 5 Center for Menopause and Reproductive Medicine Research, Kaohsiung Chang Gung Memorial Hospital, Chang Gung University College of Medicine, Kaohsiung 833, Taiwan

**Keywords:** microRNA, miR-372-3p, spent culture medium, blastocyst morphology, morphokinetics, embryo development

## Abstract

**Background/Objectives:** MicroRNAs released into spent culture medium (SCM) may reflect embryo developmental status, but their relationship with blastocyst morphology and time-lapse morphokinetic behavior remains incompletely understood. This prospective study aimed to investigate whether selected SCM microRNAs are associated with blastocyst morphological grade and early morphokinetic dynamics in human embryos. **Methods:** A total of 432 SCM samples were collected from embryos cultured in a time-lapse incubation system between October 2018 and July 2020. Blastocysts were classified as good, fair, or poor quality according to morphological grading. The expression levels of six candidate microRNAs, including miR-182-5p, miR-302a-3p, miR-372-3p, miR-373-3p, miR-518a-3p, and miR-519d-3p, were quantified using real-time quantitative polymerase chain reaction. Associations between microRNA expression, embryo morphology, and morphokinetic parameters were analyzed using GEE, multivariate logistic regression, and receiver operating characteristic (ROC) analysis. **Results:** After accounting for within-patient clustering using GEE, good-quality embryos showed significantly lower expression of miR-372-3p and miR-373-3p than poor-quality embryos. On multivariate logistic regression, lower miR-372-3p expression remained independently associated with optimal t5–t2 interval after adjustment, whereas its associations with optimal CC3 and the combined outcomes were attenuated. ROC analysis demonstrated modest discriminatory ability for miR-372-3p alone (AUC = 0.644), whereas embryo morphology alone achieved an AUC of 0.731, and the combined model incorporating morphology and miR-372-3p reached the highest AUC of 0.752. **Conclusions:** These findings suggest that SCM microRNA expression is associated with embryo development. In particular, reduced miR-372-3p expression may reflect more favorable blastocyst morphology and early developmental dynamics, supporting its potential as an adjunctive molecular indicator of embryo quality. Further validation incorporating embryo ploidy status and clinical outcomes is warranted before clinical application.

## 1. Introduction

Maximizing the likelihood of achieving a successful live birth remains the primary goal of artificial reproductive technology (ART). Traditionally, embryo selection has relied on morphological grading to prioritize embryos for transfer [1]. However, this approach does not always ensure implantation success [2]. Chromosomal abnormalities remain the leading cause of implantation failure or pregnancy loss [3]. Preimplantation genetic testing for aneuploidy (PGT-A) is commonly used to identify the chromosome status of embryos. Women of advanced maternal age or those with recurrent pregnancy loss may benefit from PGT-A [4]. Despite its growing clinical use, PGT-A has several limitations. It is an invasive procedure requiring specialized laboratory expertise, and its application may be limited when embryos demonstrate restricted developmental potential or suboptimal blastocyst quality. In addition, PGT-A provides chromosomal information but does not fully capture the dynamic molecular processes underlying embryo development. These considerations have encouraged interest in noninvasive molecular signals released by embryos into spent culture medium, which may provide additional insight into embryo developmental competence and complement conventional morphological and morphokinetic assessment [5,6].

Apart from morphological assessment at a single time point, time-lapse imaging (TLI) allows continuous monitoring of embryo development without disrupting the culture environment [7]. Embryogenesis is a dynamic process, and TLI provides objective information on developmental dynamics by enabling detailed assessment of morphokinetic parameters [8]. The timing of key developmental events, including cleavage, compaction, and blastulation, may vary considerably even among blastocysts of similar morphological grade. Abnormal developmental timing or asynchronous cell division has been associated with impaired embryo quality and reduced implantation potential [9,10]. Accordingly, several morphokinetic algorithms have been developed to characterize embryo developmental competence based on time-lapse parameters [11,12].

The analysis of spent culture medium (SCM) provides a noninvasive opportunity to investigate molecular signals released by developing embryos. Among these signals, microRNAs (miRNAs) are of particular interest because of their regulatory roles in gene expression, cell-to-cell communication, oocyte maturation, embryogenesis, implantation, and pregnancy maintenance [13]. Previous studies have demonstrated that human embryos release miRNAs into SCM, and distinct miRNA profiles have been associated with implantation potential and embryo developmental competence [14]. Cuman et al. reported that miR-661 and miR-372 levels were increased in the SCM of blastocysts that failed to implant, while Rosenbluth et al. identified differential miRNA expression in relation to implantation and aneuploidy [15,16]. As highlighted in a recent systematic review, most work in this area has relied on implantation, ploidy status, or pregnancy outcomes to define embryo competence [17], leaving the relationship between SCM miRNA expression and blastocyst morphology or early morphokinetic behavior comparatively unexplored. Among these studies, Cimadomo et al. came closest to addressing this gap, screening ten candidate miRNAs in human blastocyst SCM and identifying six miRNAs, including miR-182-5p, miR-302a-3p, miR-372-3p, miR-373-3p, miR-518a-3p, and miR-519d-3p, with significant univariate associations with implantation outcome [18]. These six miRNAs belong to biologically distinct but implantation-relevant families, including the miR-371-373 and miR-302 clusters, both implicated in stemness and pluripotency regulation, and the chromosome 19 miRNA cluster (C19MC), implicated in early pregnancy establishment. Yet even in that study, embryo morphology and day of full development were used only as statistical confounders for implantation outcome, rather than as variables of direct biological interest, leaving their relationship with these six miRNAs uncharacterized.

Therefore, this study aimed to evaluate the expression of these six previously identified miRNAs in SCM and to determine their associations with blastocyst morphology and time-lapse morphokinetic parameters. We hypothesized that SCM miRNA expression reflects embryo developmental dynamics and may serve as an adjunctive molecular indicator of embryo quality.

## 2. Materials and Methods

### 2.1. Study Population

This prospective observational cohort study investigated the association between miRNA expression in SCM and embryo developmental characteristics, including blastocyst morphology and time-lapse morphokinetic parameters. Patients who underwent in vitro fertilization (IVF) or intracytoplasmic sperm injection (ICSI) treatment at the Kaohsiung Chang Gung Memorial Hospital were prospectively recruited between October 2018 and July 2020. The controlled ovarian stimulation protocols and laboratory workflow followed the standard clinical practices that have been previously described [19]. Inclusion criteria were patients with embryos cultured to day 5 or 6 in the TLI system (CCM-iBis, ASTEC Co., Fukuoka, Japan). Embryos with unclear TLI, poor quality of miRNA extraction, and developmental arrest were excluded from analysis.

### 2.2. Embryo Management and Grading

All embryos were fertilized using conventional IVF or ICSI and then cultured for 5 or 6 days after insemination using a single embryo culture TLI system. The embryos were assessed on days 1, 3, 5, and 6. Blastocysts were classified based on their expansion and morphological appearances of the inner cell mass and the trophectoderm cells [1]. Embryos that did not undergo cell division for two consecutive 24 h periods were considered to be experiencing developmental arrest [20]. The grading system categorized embryos into “good”, “fair”, and “poor” quality. In detail, ‘good’ is defined as blastocysts of AA, AB, and BA quality; ‘fair’ is for blastocysts of BB, AC, and CA quality; and ‘poor’ is for blastocyst of CC, BC, and CB quality. Two experienced embryologists reached a consensus on the grades for the embryos using the same criteria. If there were any disagreements, a thorough discussion would be held.

### 2.3. Time-Lapse Assessment

The TLI system utilized a red LED light source with a peak wavelength of 623 nm paired with a CCD camera unit connected to a 10× biological microscope. The camera’s resolution was 1.3 megapixels, and the observed area measured 4.86 mm by 3.62 mm. Photos of each embryo were taken every 15 min at 11 focal planes separated by 5 μm. The time of cell division was recorded in hours (h) and was retrospectively reviewed. Morphokinetics parameters included the time points for reaching 2 (t2), 3 (t3), 4 (t4), and 5 (t5) cells; the duration of the second (CC2 = t3–t2) and third (CC3 = t5–t3) cell cycle; the interval t5–t2; and the synchrony of division from 2 to 4 cells (S2 = t4–t3). The predefined optimal morphokinetic intervals were selected from previously published time-lapse models and were used as developmental reference ranges in the present study. Optimal intervals were defined as CC2 between 5 and 11.9 h, S2 less than 0.76 h, CC3 between 11.7 and 18.2 h, and t5–t2 greater than 20.5 h [21,22,23]. These cutoffs were applied to examine whether SCM miRNA expression was associated with favorable developmental timing.

### 2.4. Spent Culture Medium Collection and miRNA Extraction

We used the sequential culture media for embryo culture. Embryos were cultured in G1TM medium (Vitrolife Sweden AB, Västra Frolunda, Sweden) for the first 3 days and then changed to G2TM medium (Vitrolife Sweden AB) from day 3 till day 5 or 6. The SCM was collected on day 5 or 6 before embryo transfer or freezing. To avoid cross-contamination, separate Pasteur pipettes were applied for handling each embryo. Each blastocyst spent culture media (25–30 µL) was transferred into Eppendorf tubes and frozen immediately at −80 °C for subsequent miRNA analysis.

Six miRNAs (miR-182-5p, miR-302a-3p, miR-372-3p, miR-373-3p, miR-518a-3p, and miR-519d-3p) were selected a priori based on a previous study, which reported that the expression of these miRNAs was associated with implantation [18]. No additional data-driven screening or selection was performed on the current dataset prior to analysis. MiRNA isolation was carried out using the miRNeasy Serum/Plasma Advanced Kit (Qiagen, Hilden, Germany). MiR-16-5p was used as the endogenous reference, and UniSP6 as a spike-in control. Retro-transcription was performed using the miRCURY LNA RT Kit (Qiagen, Germany) with template RNA, 5X Reaction Buffer, enzyme mix, and RNase-free water. The cDNA was stored at −20 °C until further use and was pre-diluted 1:19 with nuclease-free water. Quantitative real-time PCR (RT-qPCR) analysis followed the manufacturer’s instructions using the miRCURY LNA SYBR^®^ Green PCR Kits (Qiagen, Germany). The miRNA IDs can be found in Supplementary Table S1. The ABI 7500 Fast Real-Time PCR System (Applied Biosystems, Singapore) was used to assess miRNA expression, with amplification under the following conditions: 95 °C for 2 min; 40 cycles of 95 °C for 10 s, 56 °C for 60 s. Ct values represent the number of cycles required for the fluorescent signal to cross the threshold of detection. ΔCt values were calculated as Ct (miRNA of interest) − Ct (miR-16-5p). All miRNA measurements were performed in technical duplicate, with UniSp6 also serving as a reverse-transcription/PCR technical control. Reactions with no detectable signal within the 40-cycle run were recorded as “Undetermined” and considered undetected, with no additional Ct cutoff applied. Samples with undetected miR-16-5p and UniSP6 were excluded from analysis.

### 2.5. Prediction of miRNA Target Genes and Pathway Analysis

TargetScan Human release 7.2 website (http://www.targetscan.org/vert_72/, accessed on 13 September 2023), miRDB v6.0 (http://mirdb.org/), and miRanda (microRNA.org) were used to identify the miRNA target genes. Genes predicted by any of the three databases were included in the union set. Gene ontology (GO) and KEGG (Kyoto Encyclopedia of Genes and Genomes) enrichment analysis of the predicted genes were performed using the MultimiR v1.20.0 and ClusterProfiler v4.6.2. The GO analysis contained three categories: biological process, cellular component, and molecular function. Adjusted *p*-values < 0.05 were defined as significant. Graphs of GO and KEGG analysis were produced by ggplot2 v3.4.2.

### 2.6. Statistical Analysis

Continuous data are presented as medians and interquartile ranges (IQRs), while categorical variables are shown as numbers and percentages. Chi-square tests or Fisher’s exact tests were used to compare detection rates of six miRNAs and proportions of embryos with optimal morphokinetic intervals across the three embryo quality groups. The Kruskal–Wallis test was used to assess differences in miRNA ΔCt values and morphokinetic parameters among groups, with post hoc pairwise comparisons performed using Dunn’s test with Bonferroni correction (adjusted threshold: *p* < 0.0167). The same test was used to evaluate the stability of miR-16-5p and UniSp6 across quality groups and culture days. The Mann–Whitney U test was used to compare miRNA ΔCt values between embryos within and outside predefined optimal morphokinetic intervals, with Bonferroni correction applied across six miRNAs (adjusted threshold: *p* < 0.0083).

Since multiple embryos were obtained from the same patient, generalized estimating equations (GEE) with an exchangeable working correlation structure and robust standard errors were used to assess the association between embryo morphological grade and miRNA ΔCt values (Gaussian family, identity link). Poor-quality embryos were used as the reference category. An unadjusted model and an adjusted model incorporating maternal age and culture day were fitted.

Univariate and multivariable logistic regression analyses were performed to evaluate associations between miRNA expression, embryo morphology, and morphokinetic outcomes (optimal vs. non-optimal). As multiple embryos originated from the same patient, these analyses were similarly fitted using GEE, with poor-quality embryos as the reference category. Multivariable models were adjusted for embryo morphological grade, maternal age, and culture day. Stratified analyses by culture day were additionally performed to evaluate their influence on these associations. Three predictive models were constructed for comparison: (1) miRNA ΔCt value alone, (2) morphological grading alone, and (3) a combined model. Receiver operating characteristic (ROC) curves and area under the curve (AUC) were calculated to assess discriminative ability. All analyses were performed using SPSS version 26.0 (IBM, Armonk, NY, USA) and R version 4.3.3 (R Foundation for Statistical Computing, Vienna, Austria). A two-sided *p* < 0.05 was considered statistically significant unless otherwise specified.

## 3. Results

### 3.1. Baseline Patient and Embryo Characteristics

A total of 432 spent culture medium samples from 88 patients were included in the analysis. The mean maternal age was 34.41 ± 4.2 years. Among the included embryos, 367 (85%) samples were collected and vitrified on day 5, and 65 (15%) on day 6. According to embryo morphology, 168 embryos were classified as good quality, 138 as fair quality, and 126 as poor quality (Table 1). The distribution of D5 and D6 embryos differed significantly across morphological quality groups (χ^2^ = 42.2, df = 2, *p* < 0.0001): D6 embryos accounted for 31.7% of the poor-quality group, compared with 12.3% of the fair-quality group and 4.8% of the good-quality group. The Ct values of the endogenous reference miR-16-5p also differed significantly between D5 and D6 embryos (Mann–Whitney U, *p* < 0.0001), with lower (more abundant) miR-16-5p Ct values observed in D6 embryos.

### 3.2. MiRNA Detection and Expression Patterns According to Embryo Morphological Grade

**Figure 1:**
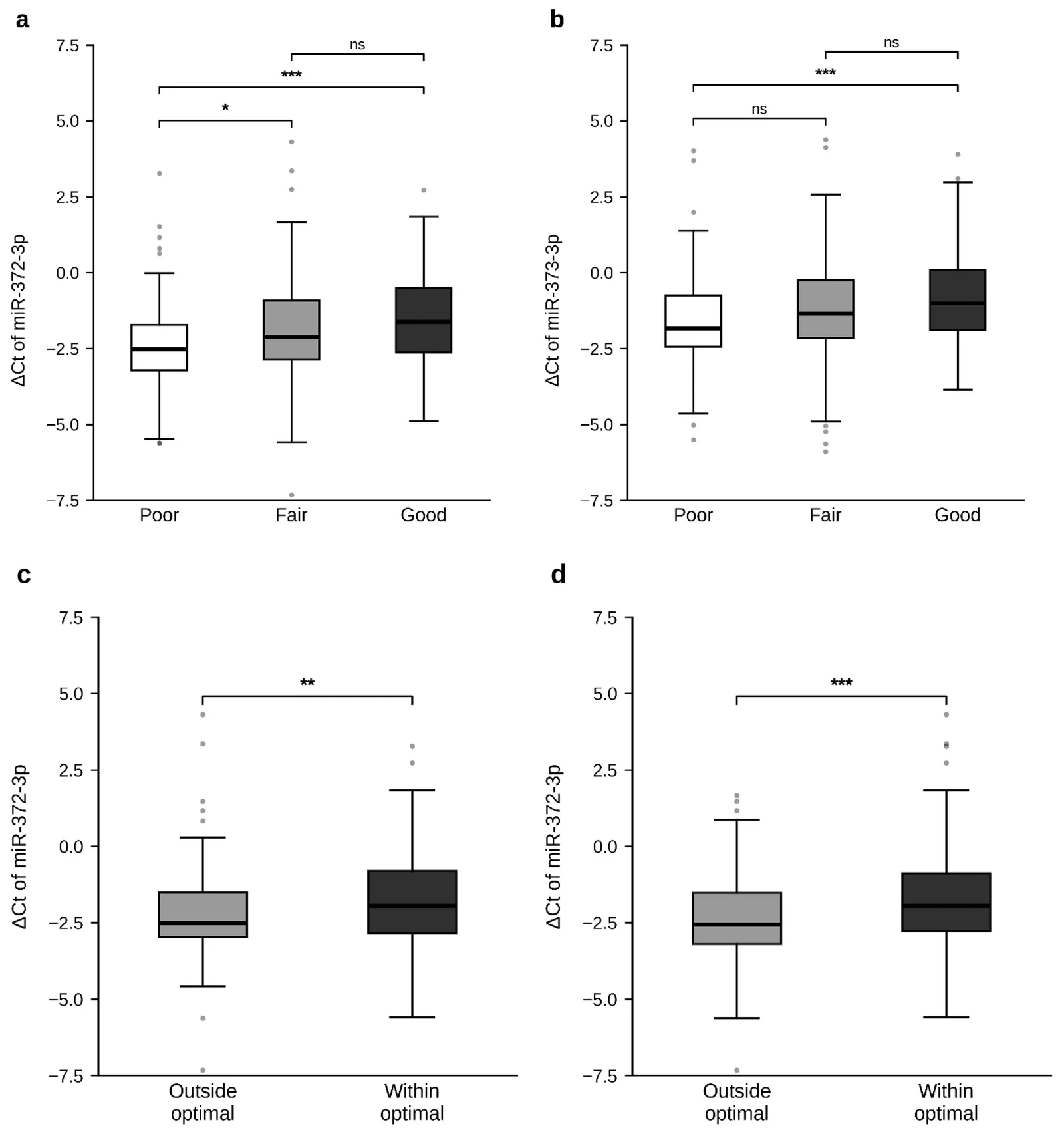
Association of SCM miRNA expression with embryo morphology and morphokinetic intervals. (**a**) Relative expression of miR-372-3p according to embryo morphology. (**b**) Relative expression of miR-373-3p according to embryo morphology. (**c**) Relative expression of miR-372-3p in embryos within and outside the optimal CC3 interval. (**d**) Relative expression of miR-372-3p in embryos within and outside the optimal t5–t2 interval. Higher ΔCt values indicate lower relative miRNA expression. * *p* < 0.05; ** *p* < 0.01; *** *p* < 0.001; ns, not significant. SCM, spent culture medium.

The detection rates and expression levels of the six selected miRNAs according to embryo morphology are summarized in Table 2. The detection rates of miR-182-5p, miR-302a-3p, and miR-518a-3p differed significantly among the three morphology groups (*p* = 0.0021, 0.0004, and 0.0001, respectively) with higher detection rates observed in poor-quality embryos than in good-quality embryos. In contrast, the detection rates of miR-372-3p, miR-373-3p, and miR-519d-3p did not differ significantly among groups (all *p* > 0.05). Regarding relative expression, the ΔCt values of miR-372-3p and miR-373-3p differed significantly across embryo morphology groups (*p* < 0.0001, and *p* = 0.0001, respectively). Good-quality embryos had higher ΔCt values, indicating lower relative expression, for miR-372-3p (*p* < 0.0001) and miR-373-3p (*p* < 0.0001) compared with poor-quality embryos (Figure 1a,b). Fair-quality embryos also showed higher ΔCt values of miR-372-3p compared with poor-quality embryos (*p* = 0.0141) (Figure 1a). The Ct values of the endogenous reference miR-16-5p differed significantly across morphological quality groups (Kruskal–Wallis, *p* < 0.0001), with lower (more abundant) miR-16-5p Ct values observed in poor-quality embryos; in contrast, the UniSp6 exogenous spike-in control showed no significant variation across quality groups (*p* = 0.65).

GEE analysis was performed for both miR-372-3p and miR-373-3p to evaluate the association between miRNA expression and embryo morphological grade. Poor-quality embryos were used as the reference category. In the unadjusted model (Supplementary Table S2), good-quality embryos had significantly higher ΔCt values than poor-quality embryos for both miR-372-3p and miR-373-3p (β = 0.80 and 0.60, respectively, both *p* < 0.0001), indicating lower expression of both miRNAs in good-quality embryos. The fair- versus poor-quality difference was significant for miR-372-3p (β = 0.42, *p* = 0.0210) but not for miR-373-3p (β = 0.28, *p* = 0.1553). After adjustment for maternal age and culture day (Table 3), the association between good-quality embryos and elevated ΔCt remained statistically significant for both miR-372-3p (β = 0.66, *p* = 0.0002) and miR-373-3p (β = 0.47, *p* = 0.0058). The fair-quality difference was attenuated and non-significant for both miRNAs following adjustment. Among the covariates, culture day was a significant predictor of both miR-372-3p and miR-373-3p (*p* = 0.0157 and 0.0470, respectively), indicating that D6 embryos had higher expression of both miRNAs regardless of morphological grade. Maternal age was additionally associated with miR-373-3p expression (*p* = 0.0382). The magnitude of the good- versus poor-quality difference was greater for miR-372-3p (β = 0.66) than for miR-373-3p (β = 0.47), suggesting stronger discriminatory potential of miR-372-3p for embryo quality.

### 3.3. Morphokinetic Characteristics According to Embryo Morphology

The distribution of predefined optimal morphokinetic intervals also differed according to embryo morphology (Table 2). The proportion of embryos within the optimal CC2 interval progressively decreased from the good- to poor-quality embryos (*p* < 0.0001). Similar patterns were observed for the CC3 and t5–t2 intervals (both <0.0001), with good-quality embryos showing higher proportions of optimal developmental timing than fair- and poor-quality embryos. No significant difference was observed for the S2 interval.

### 3.4. Association Between miRNA Expression and Morphokinetic Intervals

We next compared miRNA expression between embryos within and outside the predefined optimal morphokinetic intervals (Table 4). Embryos within the optimal CC3 interval showed lower relative expression of miR-372-3p and miR-373-3p (*p* = 0.0024 and 0.0026, respectively) (Figure 1c). Similarly, embryos within the optimal t5–t2 interval showed lower relative expression of miR-372-3p (*p* = 0.0003) (Figure 1d). No significant differences were observed for miR-182-5p, miR-302a-3p, miR-518a-3p, or miR-519d-3p across the predefined morphokinetic intervals. These findings suggest that miR-372-3p, and to a lesser extent miR-373-3p, are associated with favorable early developmental timing.

### 3.5. Logistic Regression Analysis of Optimal Morphokinetic Intervals

Logistic regression analysis was performed to further evaluate the association between miRNA expression and optimal morphokinetic intervals (Table 5). Because multiple embryos originated from the same patient, these analyses were fitted using GEE to account for within-patient clustering. In univariate analysis, higher ΔCt values of miR-372-3p (OR 1.24, 95% CI: 1.04–1.47, *p* = 0.0154) and miR-373-3p (OR 1.21, 95% CI: 1.02–1.44, *p* = 0.0297) were associated with an increased likelihood of embryos falling within the optimal CC3 interval. However, these associations were attenuated and no longer significant after adjusting for embryo morphological grade, maternal age, and culture day, whereas embryo morphological grade remained a strong independent predictor. For the t5–t2 interval, higher ΔCt values of miR-372-3p remained significantly associated with optimal developmental timing in both univariate (OR 1.32, 95% CI: 1.12–1.55, *p* = 0.0008) and multivariable analysis (OR 1.19, 95% CI: 1.01–1.41, *p* = 0.0399), independent of embryo morphological grade. When embryos were classified according to the combined outcome of both optimal CC3 and optimal t5–t2 intervals, miR-372-3p was significantly associated with this favorable morphokinetic pattern in univariate analysis (OR 1.22, 95% CI: 1.04–1.43, *p* = 0.0156), although the association was attenuated after adjustment (OR 1.05, 95% CI: 0.88–1.26, *p* = 0.6013).

### 3.6. Discriminatory Performance of miRNA Expression and Embryo Morphology

**Figure 2:**
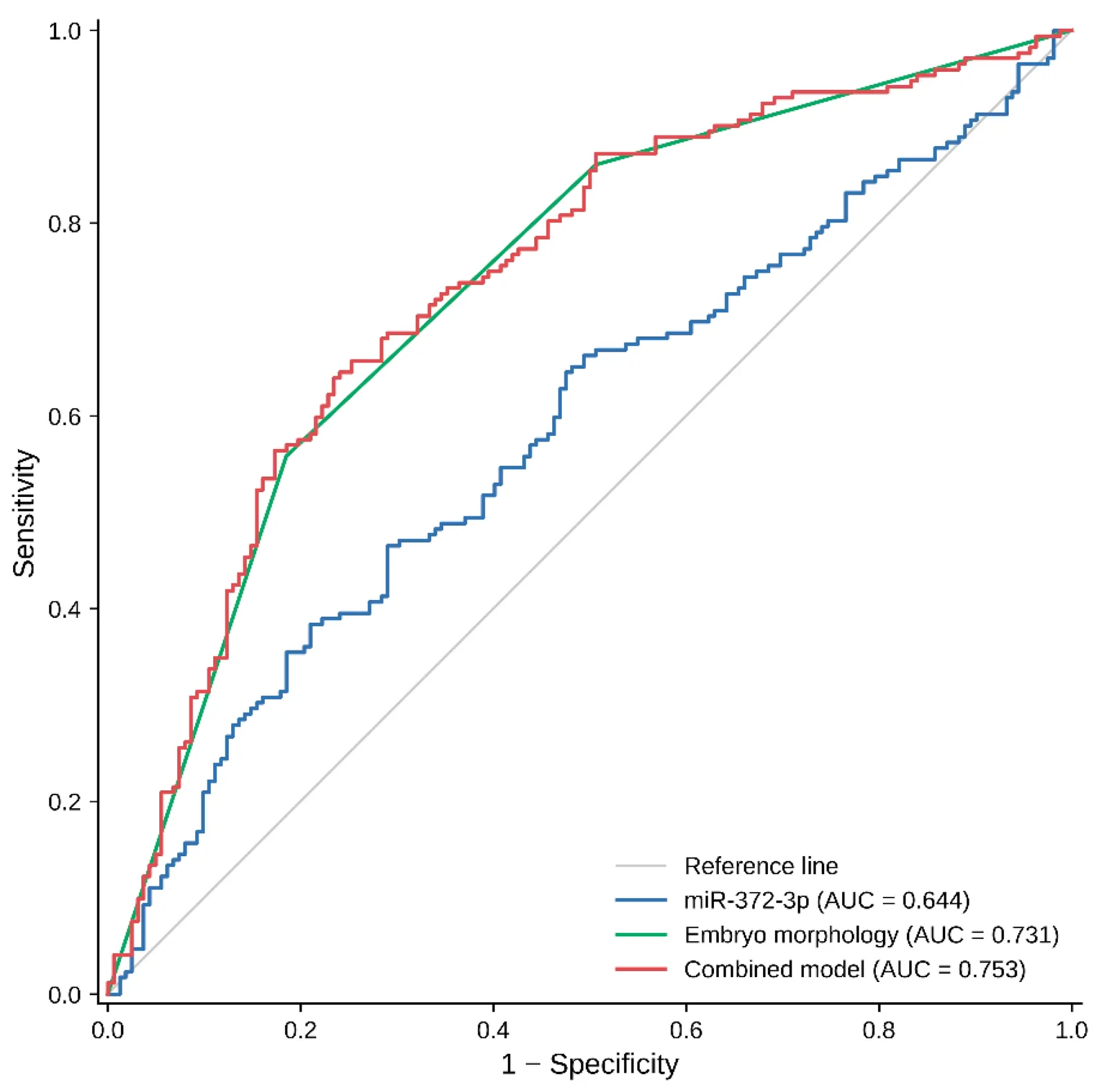
ROC curve analysis for embryos with both optimal CC3 and optimal t5–t2 intervals. ROC curves compare the discriminatory performance of miR-372-3p expression alone, embryo morphology alone, and the combined model including both miR-372-3p expression and embryo morphology. ROC, receiver operating characteristic; AUC, area under the curve.

ROC curve analysis was performed to compare the discriminatory performance of miRNA expression, embryo morphology, and their combined models for predefined morphokinetic outcomes (Figure 2 and Supplementary Figure S1). For the optimal CC3 interval, miR-372-3p and miR-373-3p alone showed modest discriminatory ability, whereas embryo morphology demonstrated superior discriminatory performance (Supplementary Figure S1a). Similar findings were observed for the optimal t5–t2 interval (Supplementary Figure S1b). For the combined outcome of both optimal CC3 and optimal t5–t2 intervals, miR-372-3p achieved an AUC of 0.644, morphological grade alone achieved an AUC of 0.731, and the combined model achieved the highest AUC of 0.752 (Figure 2). These results indicate that miR-372-3p may provide additional molecular information related to embryo developmental timing, but its discriminatory performance alone was limited.

### 3.7. Exploratory Pathway Enrichment Analysis

**Figure 3:**
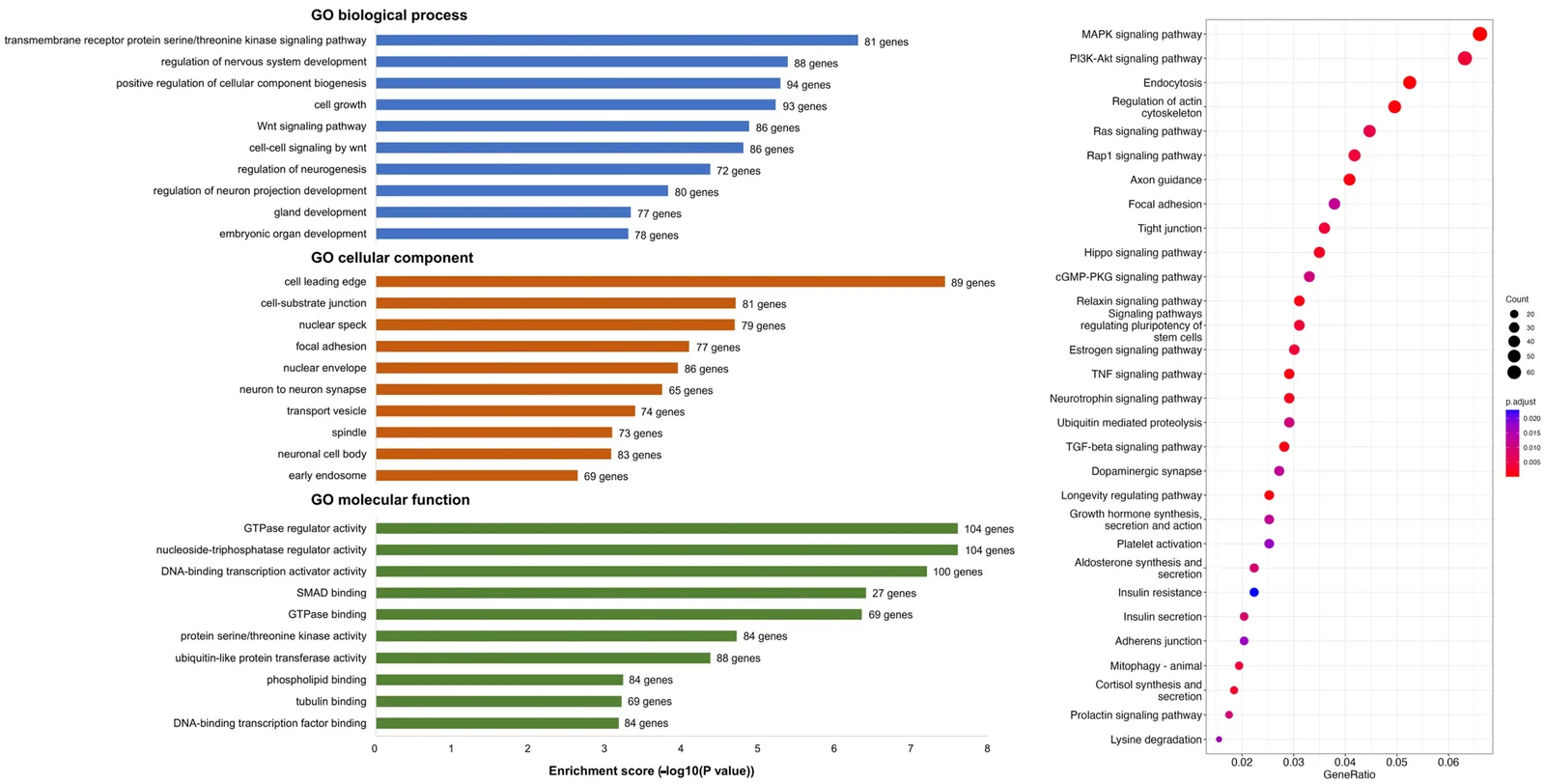
Functional enrichment analysis of predicted target genes of selected SCM miRNAs. Top 10 GO terms (ranked by enrichment score and adjusted *p*-value) in the biological process, cellular component, and molecular function categories. Top 30 significantly enriched KEGG pathways (ranked by gene ratio). GO: gene ontology, KEGG: Kyoto Encyclopedia of Genes and Genomes.

To explore the potential biological relevance of the selected miRNAs, target genes were predicted and subjected to GO and KEGG enrichment analyses. A total of 2536 target genes were identified as the union of predictions, of which 282 genes were predicted by all three databases. Enriched biological processes were mainly related to transmembrane receptor protein serine/threonine kinase signaling, regulation of nervous system development, and positive regulation of cellular component biogenesis. KEGG pathway analysis showed enrichment in pathways related to MAPK signaling, PI3K-Akt signaling, endocytosis, regulation of actin cytoskeleton, and Ras signaling (Figure 3). The complete KEGG gene lists are provided in Supplementary Table S3.

## 4. Discussion

In this prospective observational cohort study, we explored the relationship between selected miRNAs in spent culture medium and human embryo developmental characteristics, including blastocyst morphology and time-lapse morphokinetic parameters. The main finding was that miR-372-3p and miR-373-3p expression differed according to embryo morphological grade, with good-quality embryos showing lower relative expression of these miRNAs. In addition, lower miR-372-3p expression was associated with favorable morphokinetic timing, particularly optimal t5–t2 intervals. These findings suggest that miRNA expression in spent culture medium may reflect embryo developmental dynamics and provide adjunctive molecular information beyond conventional morphological observation.

In the present study, miR-372-3p and miR-373-3p expression were associated with embryo morphology and early developmental timing. Of the two miRNAs, miR-372-3p showed the most consistent association with embryo developmental characteristics. Good-quality embryos had lower relative expression of miR-372-3p, and embryos within optimal CC3 and t5–t2 intervals also showed lower miR-372-3p expression. This finding is consistent with prior experimental evidence implicating this miRNA cluster in early embryonic development. The miR-371-373 cluster is highly expressed in human embryonic stem cells and has been implicated in pluripotency, cell-cycle regulation, and early embryonic development [24]. Experimental studies in mice have shown that the murine miR-290-295 cluster, the homolog of human miR-371-373, is important for embryonic growth and germ cell development [25]. Loss of miR-290-295 expression results in reduced germ cell colonization of the gonads, lowers the blastocyst development rate, and decreases fertility. Female mice born from miR-290-295^−/−^ blastocysts exhibit sterility and premature ovarian failure. This cluster also promotes embryonic stem cell proliferation by facilitating the G1-S transition in the cell cycle [26]. Notably, several of these processes overlap with the pathways identified in our exploratory GO and KEGG enrichment analysis of predicted miR-372-3p/miR-373-3p target genes, including MAPK signaling, PI3K-Akt signaling, and regulation of the cell cycle (Figure 3), lending indirect support to a potential role for these miRNAs in regulating cell proliferation and developmental progression during preimplantation development. Taken together with our findings, these data suggest that altered miR-372-3p expression in SCM may reflect differences in embryonic cellular activity, developmental pace, or stress-related secretion patterns during preimplantation development.

The relationship between lower SCM miR-372-3p expression and favorable embryo development should be interpreted carefully. One possible explanation is that embryos with less favorable developmental competence may release higher amounts of specific miRNAs into the culture medium because of altered cellular turnover, impaired cell-to-cell communication, or increased extracellular vesicle secretion. Another possibility is that SCM miRNA levels reflect trophectoderm activity during blastocyst formation, as miRNAs in spent culture medium are thought to primarily originate from trophectoderm cells [27]. Since miRNA expression was measured only in SCM and not directly in paired embryonic cells, the cellular origin and biological meaning of these extracellular miRNAs cannot be definitively determined in the present study.

The stage-dependent nature of miRNA secretion also has implications for our study design. Capalbo et al. found that miRNA counts in cleavage- and morula-stage media were indistinguishable from negative controls, with a marked increase only at the blastocyst stage [27]. A similar stage-dependent increase has been reported in a murine model comparing zygote, 4-cell, and blastocyst-stage media [28]. This also relates to our use of sequential culture media rather than a single-step medium collected throughout the full culture course. A single-step medium would in principle allow miRNA to be tracked across the entire developmental process; however, because miRNA release has been reported to increase substantially only after the cleavage stage, the sequential-media approach used here was still able to capture the relevant window of miRNA dynamics leading up to blastocyst formation. Future studies may consider adopting single-step culture media together with a broader panel of morphokinetic parameters to more fully characterize miRNA secretion dynamics across preimplantation development. Notably, our GEE analysis identified culture day as an independent predictor of both miR-372-3p and miR-373-3p expression, with D6 embryos showing higher expression than D5 embryos regardless of morphological grade. This finding suggests that extended culture duration itself may influence miRNA release, possibly reflecting prolonged metabolic activity, cumulative cellular stress, or differences in trophectoderm cell number between D5 and D6 blastocysts. Maternal age was additionally associated with miR-373-3p expression, raising the possibility that oocyte- or embryo-intrinsic factors related to aging may also contribute to variability in SCM miRNA secretion.

The morphokinetic findings further support the concept that SCM miRNAs may be linked to embryo developmental dynamics. Consistent with previous reports, abnormal morphokinetic timing has been associated with impaired embryo developmental competence [9,29,30]. In the present study, miR-372-3p was associated with optimal t5–t2 timing even after adjustment for embryo morphology, maternal age, and day of embryo culture. However, its association with optimal CC3 and the combined CC3/t5–t2 outcome was attenuated after adjustment, especially after accounting for within-patient clustering. These results indicate that miR-372-3p may not function as an independent predictor across all morphokinetic endpoints, but rather as an adjunctive molecular indicator that partially overlaps with conventional morphological and developmental assessment. In addition, because the morphokinetic parameters examined here occur during cleavage-stage development, whereas SCM miRNA levels were measured later at the blastocyst stage, these findings should be interpreted as an association between earlier developmental kinetics and later miRNA expression rather than a concurrent relationship.

ROC analysis also supports a conservative interpretation. MiR-372-3p alone showed modest discriminatory ability for identifying embryos with favorable morphokinetic timing (AUC = 0.644), whereas embryo morphology demonstrated better performance (AUC = 0.731). The combined model including morphology and miR-372-3p achieved the highest (AUC = 0.752), but the improvement over morphology alone was limited (ΔAUC = 0.021). Internal validation of these ROC-based analyses (e.g., bootstrap resampling or cross-validation) was not performed. Therefore, these AUC values should be interpreted as exploratory estimates rather than a validated stand-alone biomarker for embryo selection. Given the additional cost, technical complexity, and turnaround time required for SCM miRNA testing, this incremental gain is unlikely to justify its routine clinical use at present. Instead, SCM miR-372-3p may provide complementary molecular information regarding embryo developmental status when integrated with morphology and time-lapse parameters.

Exploratory GO and KEGG enrichment analyses suggested that predicted target genes of the selected miRNAs were involved in pathways related to MAPK signaling, PI3K-Akt signaling, endocytosis, regulation of the actin cytoskeleton, and Ras signaling. These pathways are biologically plausible given the established roles of the miR-371-373 cluster in cell-cycle regulation and pluripotency, and may reflect broader involvement in preimplantation development, cell proliferation, and embryo–environment communication. However, these analyses were based on computationally predicted targets rather than experimentally validated target genes, and additional functional validation was beyond the scope of the current study. Therefore, the pathway results should be regarded as hypothesis-generating rather than confirmed mechanisms, and future studies incorporating target-specific validation (e.g., qPCR confirmation of predicted targets, luciferase reporter assays, or in vitro knockdown/overexpression models) are needed to establish causal roles for these miRNAs in embryonic development.

This study has several limitations. First, the low abundance of extracellular miRNAs in embryo culture medium may increase technical variability and limit assay sensitivity, despite the relatively large sample size for SCM miRNA analysis. Second, the endogenous reference miR-16-5p showed statistically significant Ct differences across quality groups and culture days, raising a legitimate concern that reference-gene instability could influence the reported effect sizes. We did not perform a formal reference-gene stability analysis or normalize against multiple endogenous reference miRNAs; consequently, the precise magnitude of the reported effect sizes should be interpreted with caution. Third, although GEE analysis was used to account for within-patient clustering, the relatively small number of patients may limit the precision of the estimated within-cluster correlation and the generalizability of the findings. Fourth, stratified sensitivity analyses showed directionally consistent associations in the D5 subset, which comprised 85% of the sample, whereas the D6 subset (*n* = 65) was underpowered to support stable multivariable inference, limiting our ability to confirm that the observed associations hold independently within each culture-day stratum. Fifth, only six candidate miRNAs, selected a priori based on prior literature, were examined; broader miRNA profiling may identify additional candidates with stronger associations with embryo development. Sixth, ploidy status was not routinely assessed and not all embryos were transferred, precluding evaluation of the relationship between SCM miRNA expression and euploidy, implantation, clinical pregnancy, or live birth outcomes. Seventh, this study did not include unconditioned blank medium, extraction, or no-template controls, limiting our ability to fully exclude environmental or reagent-related contamination as a contributor to the low-abundance signals detected. Finally, because the pathway analysis was based on predicted target genes, mechanistic conclusions cannot be drawn from these data alone.

Despite these limitations, this study provides evidence that SCM miRNA expression is associated with both blastocyst morphology and morphokinetic development. The findings support the concept that extracellular miRNAs released into embryo culture medium may reflect biological features of preimplantation embryo development. Further studies incorporating comprehensive miRNA profiling, paired embryo-cell and SCM analyses, ploidy assessment, and clinical outcome validation are needed to clarify the biological and clinical significance of SCM miRNAs.

## 5. Conclusions

In conclusion, miRNA expression in spent embryo culture medium was associated with human blastocyst morphology and time-lapse morphokinetic development. In particular, lower relative expression of miR-372-3p was observed in good-quality embryos and in embryos with favorable developmental timing, especially the optimal t5–t2 interval. Although miR-372-3p showed only modest discriminatory ability when used alone, it may provide adjunctive molecular information reflecting embryo developmental dynamics when integrated with conventional morphology and time-lapse assessment. Further validation studies incorporating embryo ploidy and clinical outcomes are required before SCM miRNA analysis can be considered for clinical embryo assessment.

## Figures and Tables

**Table 1:** Patient and embryo characteristics.

Characteristics	
SCM (*N*)	432
Patients (*N*)	88
Maternal age, mean ± SD, range (yr)	34.41 ± 4.16, 30–44
AMH, mean ± SD, range (ng/mL)	3.52 ± 2.19, 0.41–11.65
BMI, mean ± SD, range (kg/m^2^)	23.45 ± 4.23 (16.94–41.01)
Fertilization method	
IVF	77
ICSI	11
Ovarian stimulation protocol	
GnRH antagonist protocol	88
Day of SCM collection and vitrification	
5	367
6	65
Expansion of blastocysts	
Full	138
In hatching or fully hatched	48
Embryo morphological grade (day 5/6)	
Good	168 (160/8)
Fair	138 (121/17)
Poor	126 (86/40)

Note. SCM: Spent culture medium; IVF: in vitro fertilization; ICSI: intracytoplasmic sperm injection; AMH: anti-Müllerian hormone; BMI: body mass index.

**Table 2:** miRNAs Characteristics and Morphokinetic Parameters by Embryo Morphological grade.

	Good	Fair	Poor	*p*-Value
SCMs (*N*)	168	138	126	
**Detection rate, ** ***N *** **(%)**				
miR-182-5p	97 (57.7%) ^a^	86 (62.3%) ^b^	97 (77%)	0.0021 *
miR-302a-3p	115 (68.5%) ^a^	105 (76.1) ^b^	111 (88.1%)	0.0004 *
miR-372-3p	161 (95.8%)	129 (93.5%)	125 (99.2%)	0.0562
miR-373-3p	157 (93.5%)	132 (95.7%)	123 (97.6%)	0.2384
miR-518a-3p	116 (69.0%) ^a^	94 (68.1%) ^b^	111 (88.1%)	0.0001 *
miR-519d-3p	127 (75.6%)	107 (77.5%)	105 (83.3%)	0.2649
miRNA∆CT **, median (IQR)**				
miR-182-5p	2.74 (1.96)	2.76 (1.94)	2.78 (2.13)	0.8126
miR-302a-3p	0.76 (2.05)	0.71 (2.09)	0.51 (2.08)	0.0589
miR-372-3p	−1.61 (2.14) ^a^	−2.11 (1.96) ^b^	−2.52 (1.53)	<0.0001 *
miR-373-3p	−1.01 (2.00) ^a^	−1.35 (1.94)	−1.83 (1.71)	0.0001 *
miR-518a-3p	1.52 (1.60)	1.49 (1.57)	1.52 (1.25)	0.9090
miR-519d-3p	1.46 (1.86)	1.21 (1.99)	1.31 (1.75)	0.8129
**Percentage of embryos in optimal morphokinetic intervals, ** ***N *** **(%)**				
CC2 (5–11.9 h)	121 (65.8%) ^a^	79 (52.7%) ^b^	55 (38.7%)	<0.0001 *
S2 (<0.76 h)	120 (65.2%)	89 (59.3%)	66 (46.5%)	0.1340
CC3 (11.7–18.2 h)	114 (62.0%) ^a,c^	63 (42.0%) ^b^	43 (30.3%)	<0.0001 *
t5–t2 (>20.5 h)	150 (81.5%) ^a,c^	102 (68%) ^b^	66 (46.5%)	<0.0001 *

Note. ^a^: Good vs. Poor, ^b^: Fair vs. Poor, ^c^: Good vs. Fair, h: hours, * *p* < 0.0167, Bonferroni-corrected threshold for three pairwise group comparisons (α = 0.05/3).

**Table 3:** Adjusted GEE Analysis of miR-372-3p and miR-373-3p ΔCt Values According to Embryo Morphological Grade.

	miR-372-3p					miR-373-3p				
Parameter	β	SE	95% CI	Z	*p*-Value	β	SE	95% CI	Z	*p*-Value
Intercept	−2.18	0.14	−2.45, −1.91	−15.72	<0.0001	−1.40	0.14	−1.68, −1.11	−9.66	<0.0001
Fair vs. Poor	0.30	0.20	−0.09, 0.69	1.52	0.1288	0.17	0.21	−0.24, 0.58	0.81	0.4156
Good vs. Poor	0.66	0.18	0.31, 1.00	3.75	0.0002 *	0.47	0.17	0.14, 0.81	2.76	0.0058 *
Age (per year)	0.03	0.02	−0.01, 0.07	1.48	0.1384	0.04	0.02	0.00, 0.08	2.07	0.0382 *
D6 vs. D5	−0.52	0.22	−0.95, −0.10	−2.42	0.0157 *	−0.42	0.21	−0.83, −0.01	−1.99	0.0470 *

Note. Poor-quality embryos were used as the reference category. Higher ΔCt values indicate lower expression. Models were adjusted for maternal age and culture day (D5 as reference). * *p* < 0.05.

**Table 4:** MiRNAs expressions of embryo in and out of optimal morphokinetic intervals.

	miR-182-5p	miR-302a-3p	miR-372-3p	miR-373-3p	miR-518a-3p	miR-519d-3p
∆CT **, Median (IQR)**						
**CC2**						
5–11.9 h	2.92 (2.25)	0.46 (2.43)	−2.58 (1.61)	−1.70 (1.59)	1.62 (1.35)	1.48 (1.96)
Out	2.78 (1.61)	0.71 (1.79)	−2.50 (1.49)	−1.77 (1.53)	1.50 (1.33)	1.32 (1.44)
*p*-value	0.2472	0.3891	0.7630	0.6590	0.8743	0.4068
**S2**						
<0.76 h	2.87 (1.93)	0.39 (2.19)	−2.69 (1.26)	−1.90 (1.32)	1.45 (1.29)	1.18 (1.77)
Out	2.86 (2.05)	0.78 (1.98)	−2.10 (1.47)	−1.65 (1.47)	1.67 (1.41)	1.63 (1.91)
*p*-value	0.6981	0.0333	0.0164	0.2387	0.3426	0.5910
**CC3**						
11.7–18.2 h	2.81 (2.01)	0.55 (2.27)	−2.27 (1.73)	−1.64 (1.90)	1.62 (1.42)	1.57 (1.94)
Out	2.90 (1.90)	0.61 (2.17)	−2.62 (1.34)	−1.92 (0.95)	1.51 (1.39)	1.23 (1.72)
*p*-value	0.7015	0.3354	0.0024 *	0.0026 *	0.9266	0.1100
**t5–t2**						
>20.5 h	2.81 (2.02)	0.58 (2.29)	−2.45 (1.64)	−1.76 (1.73)	1.62 (1.51)	1.47 (1.80)
Out	3.20 (1.59)	0.52 (1.97)	−2.79 (1.21)	−1.90 (1.01)	1.55 (1.17)	1.42 (1.72)
*p*-value	0.1875	0.1103	0.0003 *	0.0978	0.5864	0.4788

Note. h: hours, * *p* < 0.0083, Bonferroni-corrected threshold for six pairwise group comparisons (α = 0.05/6).

**Table 5:** Univariate and multivariate logistic regression analysis predicting optimal morphokinetic intervals.

	Univariate Analysis		Multivariate Analysis	
	OR (95% CI)	*p*-Value	OR (95% CI)	*p*-Value
**CC3**				
miR-372-3p	1.24 (1.04–1.47)	0.0154 *	1.08 (0.85–1.37)	0.5464
miR-373-3p	1.21 (1.02–1.44)	0.0297 *	1.00 (0.81–1.24)	0.9891
Embryo morphological grade				
Fair vs. Poor	2.15 (1.21–3.79)	0.0085 *	1.82 (1.01–3.28)	0.0471 *
Good vs. Poor	7.72 (4.64–12.85)	<0.0001 *	5.62 (3.27–9.66)	<0.0001 *
**t5–t2**				
miR-372-3p	1.32 (1.12–1.55)	0.0008 *	1.19 (1.01–1.41)	0.0399 *
Embryo morphological grade				
Fair vs. Poor	2.71 (1.55–4.73)	0.0005 *	2.57 (1.40–4.70)	0.0022 *
Good vs. Poor	5.77 (3.25–10.25)	<0.0001 *	4.52 (2.47–8.27)	<0.0001 *
**CC3 & t5–t2**				
miR-372-3p	1.22 (1.04–1.43)	0.0156 *	1.05 (0.88–1.26)	0.6013
Embryo morphological grade				
Fair vs. Poor	3.34 (1.80–6.18)	0.0001 *	2.96 (1.56–5.62)	0.0009 *
Good vs. Poor	11.23 (6.02–20.34)	<0.0001 *	9.08 (4.82–17.12)	<0.0001 *

Note. All models were fitted using GEE to account for within-patient clustering. Multivariate analysis: adjust for age and day of embryo culture. Embryo morphological grade was categorized as good, fair and poor. * *p* < 0.05.

## Data Availability

The datasets used and analyzed during the current study are available from the corresponding author on reasonable request.

## Supplementary Materials

The following supporting information can be downloaded at: https://www.mdpi.com/article/10.3390/diagnostics16172835/s1, Figure S1. ROC curve analysis for individual optimal morphokinetic intervals; Table S1: MiRNA assay identification numbers used in this study; Table S2: Unadjusted GEE analysis of miR-372-3p and miR-373-3p ΔCt values according to embryo morphological grade; Table S3: Gene lists of the enriched KEGG pathways.
