# Supplementary material for: Spent Culture Medium miR-372-3p Is Associated with Blastocyst Morphology and Morphokinetic Dynamics in Human Embryos
Source: Diagnostics (Basel). 2026 Sep 3;16(17):2835. doi: 10.3390/diagnostics16172835 (PMC13564990; doi:10.3390/diagnostics16172835)
Supplement: Supplementary file 1 [file diagnostics-16-02835-s001.zip › diagnostics-4480842-supplementary.pdf]

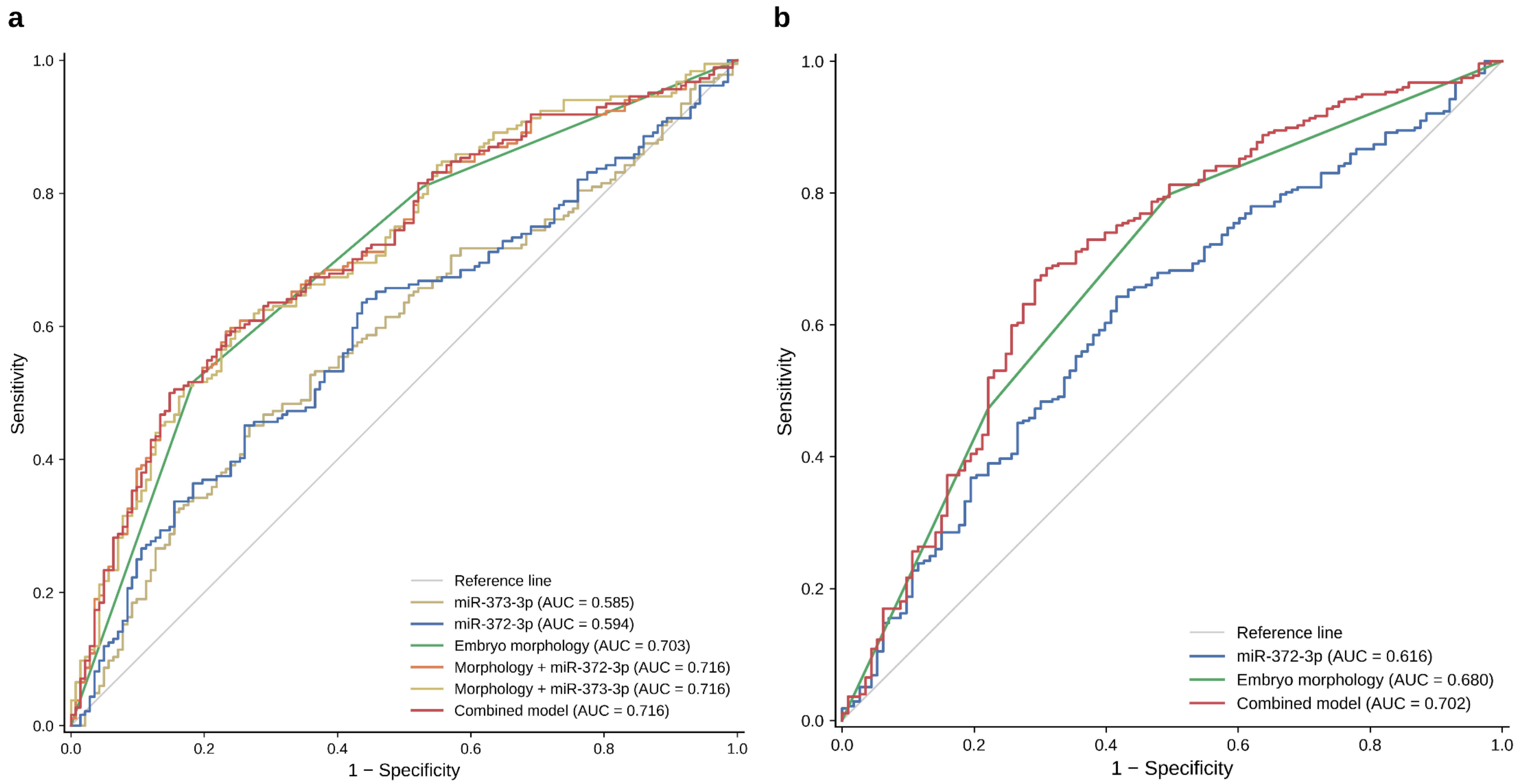

Supplementary Figure S1. ROC curve analysis for individual optimal morphokinetic intervals. (a) ROC curves for predicting embryos within the optimal CC3 interval. (b) ROC curves for predicting embryos within the optimal t5–t2 interval.

Supplemental Table S1 MiRNA assay identification numbers used in this study.

Name	Cat No	target sequence
miR-182-5p	YP00206070	UUUGGCAAUGGUAGAACUCACACU
miR-302a-3p	YP00206059	UAAGUGCUUCCAUGUUUUGGUGA
miR-372-3p	YP00204137	AAAGUGCUGCGACAUUUGAGCGU
miR-373-3p	YP00204604	GAAGUGCUUCGAUUUUGGGGUGU
miR-518a-3p	YP00206069	GAAAGCGCUUCCCUUUGCUGGA
miR-519d-3p	YP00204062	CAAAGUGCCUCCCUUUAGAGUG
miR-16-5p	YP00205702	UAGCAGCACGUAAAUAUUGGCG

All miRNA primer were used in the miRCURY LNA miRNA Focus PCR Panel (Qiagen, Germany)

Supplemental Table S2 Unadjusted GEE analysis of miR-372-3p and miR-373-3p ΔCt values according to embryo morphological grade

	miR-372-3p					miR-373-3p				
Parameter	***β***	***SE***	***95% CI***	***Z***	*p*-value	***β***	***SE***	***95% CI***	***Z***	*p*-value
**Intercept**	-2.36	0.12	-2.60, -2.12	-19.34	<0.001	-1.55	0.12	-1.79, -1.31	-12.56	<0.001
**Fair vs. Poor**	0.42	0.18	0.06, 0.77	2.31	0.0210*	0.28	0.20	-0.11, 0.66	1.42	0.1553
**Good vs. Poor**	0.80	0.16	0.49, 1.11	5.11	<0.0001*	0.60	0.15	0.30, 0.90	3.89	<0.0001*

Note. Poor-quality embryos were used as the reference category. Higher ΔCt values indicate lower expression. * $p < 0.05$

Supplemental Table S3 Gene lists of the enriched KEGG pathways

KEGG pathway	GeneRatio	BgRatio	Count	*p*.adjust	Gene lists
MAPK signaling pathway	68/1027	301/8580	68	0.000032	AKT3/MAP3K1/SOS1/ATF2/FGF1/NR4A1/MAP3K2/EREG/KITLG/PDGFRA/RASA1/RAP1A/FGF7/PPP3CB/RASGRP3/IL1RAP/MAP3K8/PGF/RELA/PRKACB/VEGFD/RAC1/CRK/ANGPT1/PDGFD/JUN/NF1/RPS6KA5/MAP3K3/PPP3CA/MAP2K7/FASLG/PPP3R1/NFKB1/MAP4K4/MAP3K5/TAOK2/DUSP2/RET/RAPGEF2/MAP4K3/TAOK3/MECOM/TGFBR2/RAP1B/CACNA1C/CACNB4/ELK4/IGF1R/RASGRF2/HSPA8/MAP3K14/RASA2/MKNK2/CACNA2D1/GRB2/MEF2C/RPS6KA4/MAPK1/MAP3K9/PAK2/MAPK9/FGF9/RPS6KA6/DUSP8/RPS6KA3/FGF5/FGF16
PI3K-Akt signaling pathway	65/1027	359/8580	65	0.003562	AKT3/ITGB3/MYB/SOS1/ATF2/FGF1/PPP2R1B/NR4A1/EREG/KITLG/PDGFRA/GNB5/FGF7/LPAR4/IL6/ITGAV/COL1A1/PGF/RELA/PRKAA1/TSC1/YWHAG/VEGFD/RAC1/ANGPT1/PDGFD/PPP2R2A/PIK3AP1/COL4A1/THBS2/CCNE2/FASLG/ATF6B/NFKB1/RBL2/MCL1/TNC/COL6A5/JAK1/MAGI1/CREB3L1/RET/MTCP1/PPP2R3A/LAMA3/PIK3CA/FOXO3/ITGB8/CCND1/IGF1R/ITGA4/CHRM2/CREB1/PRKAA2/GRB2/MAPK1/OSM/PIK3R1/CREB5/THBS1/CDK6/FGF9/YWHAE/FGF5/FGF16
Endocytosis	54/1027	250/8580	54	0.000625	ARPC1A/ARF3/PDGFRA/VPS4B/RAB4A/ARPC5/SNX32/RABEP1/IQSEC2/PIP5K1B/ACTR2/PSD/AP2B1/DAB2/SMAP1/EEA1/RAB10/PRKCI/ASAP1/ARAP2/RAB5B/SNX4/RAB11FIP5/SMAP2/ARF4/PDCD6IP/KIF5A/MVB12B/VPS26A/CHMP2B/RAB11A/RAB5A/TGFBR2/TSG101/RAB5C/ZFYVE9/RUFY2/RAB22A/IGF1R/HSPA8/GIT2/CHMP1A/WASL/NEDD4L/LDLRAP1/SH3KBP1/LDLR/RAB11FIP1/VPS26B/PSD3/EPS15L1/RAB35/SH3GLB2/CHMP1B
Regulation of actin cytoskeleton	51/1027	229/8580	51	0.000625	AKT3/ITGB3/SOS1/DIAPH2/FGF1/SSH2/ARPC1A/PDGFRA/PFN2/FGF7/RDX/LPAR4/ITGAV/PAK6/ARPC5/MYL12B/ROCK1/RAC1/CRK/MYH11/PIP5K1B/PIP4K2A/PDGFD/ACTR2/ARHGEF7/TIAM1/CFL2/PAK5/MSN/PIK3CA/NCKAP1/SPATA13/ITGB8/ITGA4/CHRM2/ROCK2/SSH1/WASL/LIMK1/DIAPH3/APC/DOCK1/MAPK1/MYLK/PIK3R1/PAK2/FGF9/CFL1/PFN1/FGF5/FGF16
Ras signaling pathway	46/1027	236/8580	46	0.004800	AKT3/SOS1/FGF1/KITLG/SHOC2/PDGFRA/GNB5/RASA1/RAP1A/FGF7/RASGRP3/PAK6/RAPGEF5/PGF/RELA/PRKACB/VEGFD/RAC1/ANGPT1/PDGFD/NF1/RAB5B/FASLG/TIAM1/NFKB1/SHC4/PAK5/RGL1/RAB5A/RAP1B/PIK3CA/RAB5C/IGF1R/RASGRF2/RASA2/GAB1/GRB2/ABL2/MAPK1/PIK3R1/PAK2/RALGDS/MAPK9/FGF9/FGF5/FGF16
Rap1 signaling pathway	43/1027	210/8580	43	0.003457	AKT3/ITGB3/FGF1/KITLG/PLCB1/PDGFRA/RAP1A/PFN2/PLCB4/FGF7/ADCY2/LPAR4/RASGRP3/RAPGEF5/PGF/VEGFD/RAC1/CRK/ANGPT1/MAGI3/PDGFD/PRKCI/APBB1IP/TIAM1/ADCY6/PRKD1/MAGI1/RAPGEF2/RAPGEF4/RAP1B/PIK3CA/IGF1R/GNAQ/DOCK4/MAPK1/PIK3R1/THBS1/RALGDS/GNAO1/FGF9/PFN1/FGF5/FGF16
Axon guidance	42/1027	182/8580	42	0.000782	EPHB1/SSH2/EPHA4/RASA1/BOC/PPP3CB/PAK6/BMPR1B/MYL12B/ROCK1/RAC1/ABLIM3/SEMA3C/L1CAM/RGMA/PPP3CA/PPP3R1/CFL2/PAK5/SRGAP2/NTN4/DCC/DPYSL2/SEMA7A/NTNG1/SEMA4B/PIK3CA/EPHA5/DPYSL5/ROCK2/EPHA7/SSH1/FZD3/LIMK1/BMPR2/WNT5A/SRGAP1/MAPK1/PIK3R1/PAK2/PLXNA1/CFL1
Focal adhesion	39/1027	203/8580	39	0.012357	AKT3/ITGB3/SOS1/PDGFRA/RAP1A/ITGAV/COL1A1/PAK6/MYL12B/PGF/ROCK1/VEGFD/RAC1/CRK/PIP5K1B/PDGFD/JUN/COL4A1/THBS2/SHC4/TNC/COL6A5/PAK5/RAP1B/LAMA3/PIK3CA/ITGB8/CCND1/IGF1R/ITGA4/ROCK2/DOCK1/GRB2/MAPK1/MYLK/PIK3R1/THBS1/PAK2/MAPK9
Tight junction	37/1027	170/8580	37	0.003087	MAP3K1/PPP2R1B/ARPC1A/RAP1A/ARHGEF2/RDX/ARPC5/MYL12B/PRKACB/PRKAA1/ROCK1/RAC1/MYH11/ACTR2/JUN/PPP2R2A/TJP2/PRKCI/MAP2K7/TIAM1/MAP3K5/RAP2C/MAGI1/CLDN1/MPP4/RAPGEF2/MSN/MPDZ/CTTN/CCND1/ROCK2/PRKCE/NEDD4L/PRKAA2/ARHGEF18/RAB8B/MAPK9
Hippo signaling pathway	36/1027	157/8580	36	0.001612	LIMD1/FGF1/LEF1/PPP2R1B/CRB1/BMPR1B/TEAD3/BIRC5/FBXW11/TCF7L1/YWHAG/PPP2R2A/PRKCI/AJUBA/GDF6/SNAI2/FRMD6/FZD8/SMAD7/SOX2/TGFBR2/ID2/LATS2/SMAD1/TCF7L2/FZD6/CCND1/FZD3/APC/BMPR2/WNT5A/MOB1B/SMAD4/CTNNA3/TP73/YWHAE
cGMP-PKG signaling pathway	34/1027	167/8580	34	0.009765	ATP1A2/AKT3/ATF2/PLCB1/EDNRB/PLCB4/ADCY2/PPP3CB/KCNMA1/ADRA1D/ROCK1/ATP1B3/VDAC1/ADRB2/PPP3CA/PPP3R1/ATF6B/ADCY6/CREB3L1/ITPR1/CACNA1C/ATP1B1/ADRA2C/GNAQ/ROCK2/PRKCE/PDE3B/CREB1/GUCY1A1/MEF2C/MAPK1/MYLK/CREB5/KCNJ8

Signaling pathways regulating pluripotency of stem cells	32/1027	143/8580	32	0.003457	AKT3/LEFTY2/BMI1/BMPR1B/STAT3/PCGF3/HESX1/NEUROG1/JAK1/ACVR1/ACVR1B/FZD8/SOX2/ID2/LEFTY1/SMAD1/PIK3CA/FZD6/REST/IGF1R/FZD3/APC/BMPR2/WNT5A/GRB2/SKIL/MAPK1/PIK3R1/SMAD5/IL6ST/INHBB/SMAD4
Relaxin signaling pathway	32/1027	129/8580	32	0.001010	AKT3/SOS1/ATF2/INSL5/PLCB1/EDNRB/GNB5/PLCB4/ADCY2/COL1A1/RELA/PRKACB/VEGFD/JUN/COL4A1/MAP2K7/ATF6B/NFKB1/ADCY6/MMP2/SHC4/EDN1/CREB3L1/TGFBR2/PIK3CA/CREB1/GRB2/MAPK1/PIK3R1/CREB5/MAPK9/GNAO1
Estrogen signaling pathway	31/1027	137/8580	31	0.003457	AKT3/SOS1/ATF2/GRM1/PLCB1/PLCB4/ADCY2/PRKACB/JUN/NCOA3/GABBR2/FKBP5/HBEGF/ATF6B/ADCY6/MMP2/SHC4/NCOA1/EBAG9/CREB3L1/ITPR1/PIK3CA/HSPA8/GNAQ/CREB1/GRB2/MAPK1/PIK3R1/CREB5/GNAO1/KCNJ6
Ubiquitin mediated proteolysis	30/1027	142/8580	30	0.009820	MAP3K1/UBR5/CDC23/ELOC/FBXW7/SAE1/RPS27A/FBXW8/FBXW11/SOCS1/KLHL13/UBE2QL1/MID1/UBE2J1/BIRC6/UBE2B/CUL5/UBE2L3/NHLRC1/ERCC8/TRIM37/RHOBTB1/UBE2A/UBE2Q2/NEDD4L/UBE3C/UBE2W/UBE2K/UBE2F/UBC
Neurotrophin signaling pathway	30/1027	119/8580	30	0.001169	AKT3/MAP3K1/SOS1/RAP1A/RELA/RAC1/CRK/JUN/ARHGDIA/RPS6KA5/MAP3K3/MAP2K7/FASLG/NFKB1/MAP3K5/SHC4/PRDM4/RAP1B/PIK3CA/FRS2/FOXO3/GAB1/GRB2/MAPK1/PIK3R1/MAPK9/RPS6KA6/TP73/YWHAE/RPS6KA3
TNF signaling pathway	30/1027	114/8580	30	0.000782	AKT3/ATF2/CFLAR/IL6/MAP3K8/RELA/VEGFD/CCL2/JUN/RPS6KA5/MAP2K7/ATF6B/NFKB1/MAP3K5/EDN1/BAG4/CREB3L1/IL15/PIK3CA/MAP3K14/CREB1/CXCL6/RPS6KA4/MAPK1/IRF1/PIK3R1/CYLD/CREB5/MAPK9/CXCL1
TGF-beta signaling pathway	29/1027	108/8580	29	0.000782	PPP2R1B/LEFTY2/RBL1/BMPR1B/ROCK1/BAMBI/RGMA/SMAD6/FBN1/GDF6/RGMB/ACVR1/ACVR1B/E2F5/SMAD7/TGFBR2/ID2/LEFTY1/SMAD1/ZFYVE9/BMPR2/SKIL/MAPK1/SMAD5/THBS1/INHBB/SMAD4/NBL1/MICOS10-NBL1
Dopaminergic synapse	28/1027	132/8580	28	0.012298	AKT3/ATF2/PPP2R1B/PLCB1/GNB5/PLCB4/PPP3CB/PRKACB/PPP2R2A/SLC18A2/PPP3CA/ATF6B/GRIA3/KIF5A/SCN1A/CREB3L1/DRD1/ITPR1/CLOCK/BMAL1/PPP2R3A/CACNA1C/GNAQ/CREB1/CREB5/MAPK9/GNAO1/KCNJ6
Platelet activation	26/1027	124/8580	26	0.017092	AKT3/ITGB3/PLCB1/RAP1A/PLCB4/ADCY2/COL1A1/MYL12B/PRKACB/ROCK1/PRKCI/APBB1IP/ADCY6/ORAI1/LYN/ITPR1/RAP1B/PIK3CA/GNAQ/ROCK2/GUCY1A1/MAPK1/MYLK/PIK3R1/SNAP23/TBXA2R
Growth hormone synthesis, secretion and action	26/1027	120/8580	26	0.012357	AKT3/MAP3K1/SOS1/ATF2/PLCB1/PLCB4/ADCY2/PRKACB/SOCS1/CRK/STAT3/SOCS2/ATF6B/ADCY6/SHC4/CREB3L1/ITPR1/CACNA1C/PIK3CA/GNAQ/CREB1/GRB2/MAPK1/PIK3R1/CREB5/MAPK9
Longevity regulating pathway	26/1027	89/8580	26	0.000625	AKT3/ATF2/ADCY2/ATG101/RELA/PRKACB/PRKAA1/TSC1/ATG5/PPARG/ATF6B/NFKB1/ADCY6/ADIPOR1/CREB3L1/PIK3CA/FOXO3/IGF1R/RB1CC1/CREB1/SESN3/APPL1/PRKAA2/ULK1/PIK3R1/CREB5
Insulin resistance	23/1027	108/8580	23	0.022499	AKT3/IL6/RELA/PRKAA1/PPP1R3C/TBC1D4/STAT3/PPP1R3B/OGT/SLC2A4/NFKB1/CREB3L1/MLX/PIK3CA/PRKCE/CREB1/PRKAA2/PIK3R1/CREB5/MAPK9/RPS6KA6/PPARA/RPS6KA3
Aldosterone synthesis and secretion	23/1027	98/8580	23	0.009017	ATP1A2/ATF2/NR4A1/PLCB1/PLCB4/ADCY2/PRKACB/NR4A2/ATP1B3/ATF6B/ADCY6/PRKD1/ORAI1/CREB3L1/ITPR1/CACNA1C/ATP1B1/GNAQ/PRKCE/CREB1/KCNK9/LDLR/CREB5
Adherens junction	21/1027	93/8580	21	0.016968	LEF1/SSX2IP/RAP1A/MYL12B/TCF7L1/ROCK1/RAC1/WASF3/YES1/SNAI2/CDH5/TGFBR2/RAP1B/TCF7L2/IGF1R/ROCK2/WASL/HEG1/MAPK1/SMAD4/CTNNA3
Insulin secretion	21/1027	86/8580	21	0.008785	ATP1A2/ATF2/RYR2/PLCB1/PLCB4/ADCY2/KCNMA1/PRKACB/KCNN2/ATP1B3/ATF6B/ADCY6/KCNN3/CREB3L1/RAPGEF4/CACNA1C/ATP1B1/GNAQ/CREB1/CREB5/VAMP2
Mitophagy - animal	20/1027	72/8580	20	0.003232	MITF/MFN2/RPS27A/RELA/E2F1/JUN/MAP1LC3B/ATG5/BNIP3L/TFE3/HIF1A/CITED2/FOXO3/BNIP3/ULK1/SQSTM1/MAPK9/BCL2L13/TBC1D17/UBC
Cortisol synthesis and secretion	19/1027	65/8580	19	0.002455	ATF2/NR4A1/PLCB1/PLCB4/ADCY2/PRKACB/KCNK2/ATF6B/ADCY6/ORAI1/CREB3L1/PDE8A/ITPR1/CACNA1C/GNAQ/CREB1/LDLR/CREB5/NCEH1
Prolactin signaling pathway	18/1027	70/8580	18	0.009675	AKT3/SOS1/RELA/SOCS1/SOCS5/STAT3/SOCS2/NFKB1/SHC4/TNFSF11/PIK3CA/FOXO3/CCND1/GRB2/MAPK1/IRF1/PIK3R1/MAPK9
Lysine degradation	16/1027	63/8580	16	0.015976	KMT2E/KMT2A/EZH1/KMT5B/DLD/NSD1/AASS/TMLHE/SUV39H2/PRDM2/MECOM/KMT2B/PRDM6/SUV39H1/PRDM16/SETD7
